# Statistical Modelling of Photonic Crystal Fibre Based Surface Plasmon Resonance Sensors Resonant Peak Wavelength for Tolerance Studies

**DOI:** 10.3390/s21196603

**Published:** 2021-10-03

**Authors:** Samuel Osifeso, Suoda Chu, K. Nakkeeran

**Affiliations:** 1 School of Engineering, Fraser Noble Building, University of Aberdeen, Aberdeen AB24 3UE, UK; s.osifeso.19@abdn.ac.uk; 2 Department of Pure and Applied Chemistry, University of Strathclyde, Glasgow G1 1XL, UK; suoda.chu@strath.ac.uk

**Keywords:** resonant peak wavelength, tolerance, design of experiments, hollow-core, photonic crystal fibre, surface plasmon resonance, refractive index, sensor, finite element method, metal coating

## Abstract

We report a statistical approach to model the resonant peak wavelength (RPW) equation(s) of a photonic crystal fibre (PCF)-based surface plasmon resonance (SPR) sensors in terms of the PCF structural parameters (air-hole diameter, pitch, core diameter and gold layer thickness) at various tolerance levels. Design of experiments (statistical tool) is used to investigate the role played by the PCF structural parameters for sensing performance evaluation—RPW, across three tolerance levels (±2%, ±5% and ±10%). Pitch of the hollow-core PCF was discovered to be the major influencing parameter for the sensing performance (RPW) of the PCF-based SPR sensor while the inner metal (gold) layer thickness and core diameter are the least contributing parameters. This novel statistical method to derive the sensing performance parameter(s) of the PCF-based SPR sensors can be applied effectively and efficiently in the designing, characterisation, tolerance analysis not only at the research level, but also in optical fibre sensor fabrication industry to improve efficiency and lower cost.

## 1. Introduction

Hollow-core photonic crystal fibres (HC-PCFs) are types of photonic crystal fibres (PCFs) that offer enormous promise for sensing applications because of the direct interaction of light and the gaseous or liquid analyte within the hollow fibre core. The periodic cladding structure of these PCFs gives them a photonic band-gap that restricts light inside the hollow-core or core filled with analytes having lower refractive index (RI). A key advantage of PCFs is that optical characteristics, such as confinement loss, dispersion and non-linearity can be influenced by varying their structural parameters [1,2,3,4]. The air-holes in the cladding can also be filled with biological and chemical analytes in gaseous or liquid forms. These are the sensing samples through which several chemical, physical and biological entities, such as temperature, pressure, liquid levels and composition (compounds and micro-organisms) are being sensed, quantified, calibrated for measurements and monitoring [5]. The sensing performance of HC-PCF-based surface plasmon resonance (SPR) sensors across these samples can be described in terms of the resonant peak wavelength (RPW) shifts, confinement loss from each sensing analyte, linearity and resolution of the sensors.

Recent studies have been reported on filling of air-holes for different SPR sensing applications. A HC-PCF-based SPR sensor was proposed by Luan and Yao [6] for SPR sensing of analytes within an RI range of 1.33 to 1.5 with sensitivity of 1800 nm/RIU. Liu et al. [7] presented a new kind of SPR sensor based on silver-coated hollow fibre structure for the detection of liquids with high RI. The highest sensitivity achieved from this PCF-SPR sensor was 6607 nm/RIU. Sensitivities of HC-PCF-based SPR sensors are measured using the wavelength interrogation method which is defined as ratio of change in the RPWs calculated between analytes to the RI change of these analytes [8]. The SPR phenomenon always occurs in HC-PCF sensors at the RPW [2]. This is the wavelength at which the real part of the effective refractive indices ($n_{\mathrm{eff}}$) of fundamental core mode and surface plasmon mode are reaching the same value [4]. This wavelength is very sensitive to the RI of the sensing analytes and their variations, hereby causing blue or red shifts to the confinement loss spectra obtained from the PCF-based SPR sensor. PCF structure influences the RPW and its shifts across various analytes with different RIs is an important sensing technique in determining the reliability of HC-PCF-based SPR sensors for RI-based detection.

Over the years, fabrication techniques, such as capillary stacking [9], drilling [10,11], sol-gel casting [12] and extrusion [13,14] have been used to achieve various PCF structures [15]. Several studies have reported the fabrication techniques of the PCFs [16,17,18,19]. However, during the fabrication of PCFs, variations from the designed values could happen to their structural parameters [20,21,22], such as air-hole diameter, pitch (that is the distance between two air-holes), core diameter and plasmonic metal layer thickness. This could either improve or deteriorate the sensing behaviour of the sensor due to the structural parameters influence discussed before. Moreover, it is good to evaluate the stability of the RPW for a HC-PCF-based SPR sensor with different tolerance levels of the variations of the PCF-SPR sensor structural parameters. In the literature [4,23,24,25], a brute-force method was typically used to investigate the tolerance level of the variations in the parameters on the sensing performances of the PCF-SPR sensors. This method lacks the ability to analyse the relationship between two or more parameters because of its one-parameter-at a-time optimisation and also results in high cost of time and computational analysis for finite element method (FEM) simulations. To overcome this issue, through this research work, we report the effective and efficient applications of the design of experiments (DoE) statistical methods introduced in the early 1950s by Box and Wilson [26], to investigate the simultaneous influence of structural parameters on the sensing performance of a HC-PCF-based SPR sensor. Factorial and Response Surface Methodology are the two types of DoE statistical tools considered for this research. Using these tools, we model the linear and then quadratic model equations for the RPW of the proposed HC-PCF-based SPR sensors in terms of the PCF structural parameters (air-hole diameter, pitch, gold layer thickness and core diameter) at various tolerance levels.

In this paper, a combined strategy with FEM simulations and statistical methods of DoE for tolerance study on a HC-PCF-based SPR sensor is proposed. To ascertain the fabrication tolerance limit possible in order to achieve stable and accurate sensing performance of a HC-PCF-based SPR sensor, this research will use the DoE statistical procedure to investigate the rate at which the RPW shifts for analytes with RI = 1.45 at higher fabrication tolerance levels. We hope that the use of DoE statistical modelling of sensing performance parameters would find wider applications in the design and optimisation of PCF sensors used for applications, such as alcohol sensing, tuberculosis cells sensing, cancer cell detection, toxic waste monitoring, etc.

## 2. Structure Design and Numerical Method

A cross-sectional structure of a HC-PCF-based SPR sensor similar to the structural design in [2] is presented for sensing analytes within the RI range of 1.43 to 1.49. In this proposed HC-PCF-based SPR sensor design, the surfaces of two air-holes adjacent to the hollow-core are coated with gold layers which are to be used for surface plasmon generation. The use of gold as the plasmonic material is to reduce confinement losses in the sensor when operating within the near-infrared range. The selected air-holes alongside the hollow-core are filled with the sensing analyte. They serve as the sensing channels for this sensor structure. The structural design of the sensor is initially set as follows: air-hole diameter d=1.4 μm, pitch Λ=2 μm, gold layer thickness $t_{g}=50\;nm$, selected air-hole diameter $d_{s}=d-t_{g}=1.35\;\mu m$, hollow-core diameter $d_{c}=1.8\;\mu m$.

**Figure 1:**
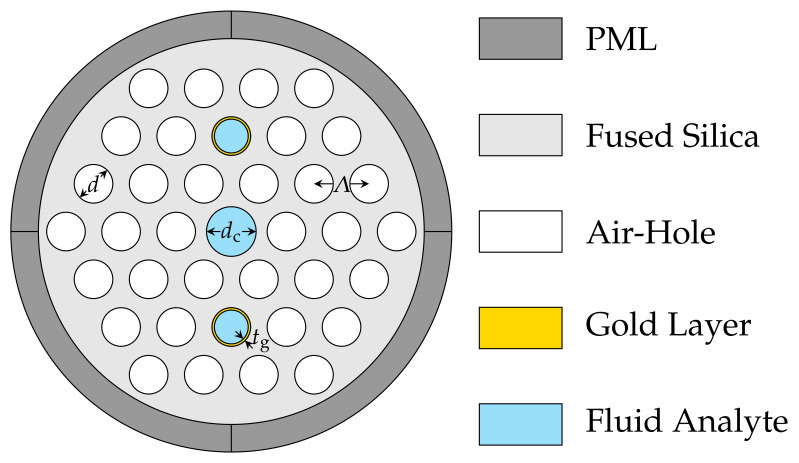
Schematic structure of the cross-section of the HC-PCF-based SPR sensor with Λ=2 μm, d=1.4 μm, $t_{g}=50\;nm$ and $d_{c}=1.8\;\mu m$.

The schematic cross-section of the proposed HC-PCF-based SPR sensor is illustrated in Figure 1. The sensing performance of the proposed sensor was analysed by FEM through COMSOL Multiphysics software and the obtained results are analysed for a RI range of 1.43 to 1.49.

Figure 1 shows the air-holes are arranged in a hexagonal lattice. The entire fibre cross-section radius was considered to be 9 μm. A perfectly matched layer (PML) of thickness 2 μm was added to the sensor structure design to absorb incident radiations without producing any back reflections [4]. The fused silica was used as the background material in this HC-PCF-based SPR sensor and its wavelength-dependent RI equation was calculated by the Sellmeier equation [27]

(1) $$n_{{\mathrm{SiO}}_{2}}\left(\lambda \right)=\sqrt{1+\frac{a_{1}\lambda^{2}}{\lambda^{2}-b_{1}^{2}}+\frac{a_{2}\lambda^{2}}{\lambda^{2}-b_{2}^{2}}+\frac{a_{3}\lambda^{2}}{\lambda^{2}-C_{3}^{2}}}$$

where $n_{{\mathrm{SiO}}_{2}}$ is the RI of the fused silica, λ is the incident light wavelength and it is measured in μm. The Sellmeier coefficients are $a_{1}$, $a_{2}$, $a_{3}$, $b_{1}$, $b_{2}$, $b_{3}$ and their values are 0.691663, 0.4079426, 0.8974794, 0.004679148 μm${}^{2}$, 0.01351206 μm${}^{2}$, and 97.93400025 μm${}^{2}$, respectively. Gold is used as the plasmonic metal layer and its dielectric function is derived from the following Drude model:

(2) $$\varepsilon \left(\lambda \right)=1-\frac{\lambda^{2}\lambda_{c}}{\lambda_{p}^{2}\left(\lambda_{c}+i\lambda \right)}$$

where $\lambda_{p}$ is the plasma wavelength of 0.16826 μm and $\lambda_{c}$ is the collision wavelength of 8.9342 μm for gold (Au) [28]. The confinement loss obtained at the RPW is derived as,

(3) $$\alpha_{\mathrm{loss}}\;\left[dB/cm\right]=8.686\times \frac{2\pi }{\lambda }ℑ\left(n_{\mathrm{eff}}\right)\times 10^{4},$$

where $\alpha_{\mathrm{loss}}$ is the confinement loss while $ℑ(n_{\mathrm{eff}})$ is the imaginary part of the effective refractive index of the fundamental core mode. The analyte RI range investigated is from 1.43 to 1.49. The sensor could be applied for detection on lymphocyte ($n_{a}$ = 1.43 ± 0.05) [29], monocytes ($n_{a}$ = 1.43 ± 0.04) [29], aqueous enzymatic extracted algae oil ($n_{a}$ = 1.445) [30], adsorbed single-stranded DNA layers ($n_{a}$ = 1.46) [31], poly-acrylic acid ($n_{a}$ = 1.47) [32] and poly-allylamine hydrochloride ($n_{a}$ = 1.49) [32].

## 3. FEM Simulation Results and SPR Discussion

**Figure 2:**
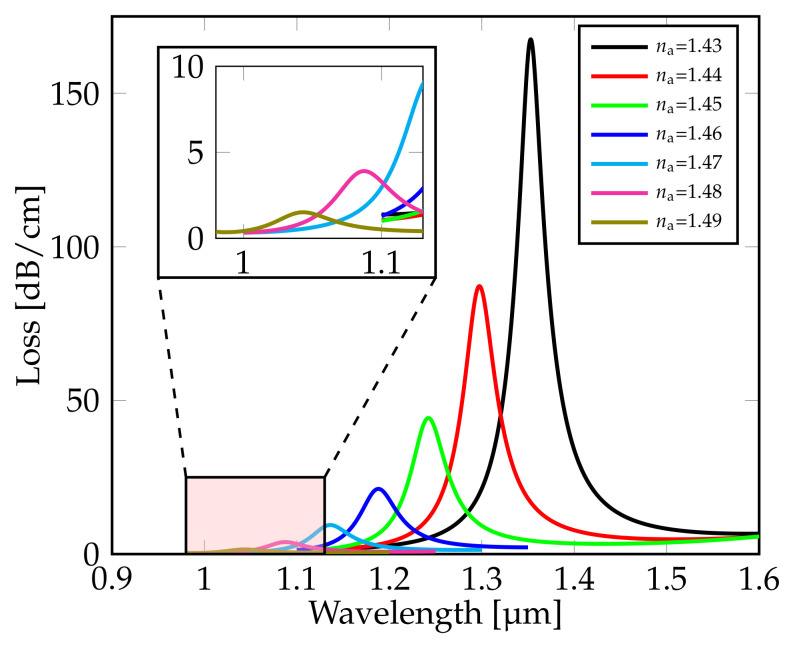
Confinement loss spectra of analyte with different RI $n_{a}$ in the range from 1.43 to 1.49.

The sensing performance of this HC-PCF-based SPR sensor was evaluated in terms of the sensitivity, which is proportional to the wavelength change of resonance peaks for two different analytes ($\Delta \lambda_{\mathrm{RPW}}$). The sensitivity is calculated with the formula

(4) $$\mathrm{Sensitivity}=\frac{\Delta \lambda_{\mathrm{RPW}}}{\Delta n_{a}}$$

where $\Delta n_{a}$ is the minimum RI change of analyte. Figure 2 shows the confinement loss spectra characteristics against wavelength range from 0.95 μm to 1.6 μm for sensing analyte with different RIs varying from 1.43 to 1.49. Results show that an increase in the RI of the analyte causes the RPW to steadily shift to lower wavelengths. As a result of the RPW shifting to shorter wavelengths when the RI of the analyte is changed from 1.43 to 1.49, it can be noticed that the confinement peak loss in the core mode steadily reduces too as shown in Table 1.

Results show that for analytes with RI = 1.45, the surface plasmons were excited from the gold metal layers at a wavelength of 1244 nm. This is due to the coupling between the fundamental core mode and plasmonic mode at that wavelength. During this process, maximum incident light energy is transferred from the fundamental core mode to the plasmonic mode.

**Figure 3:**
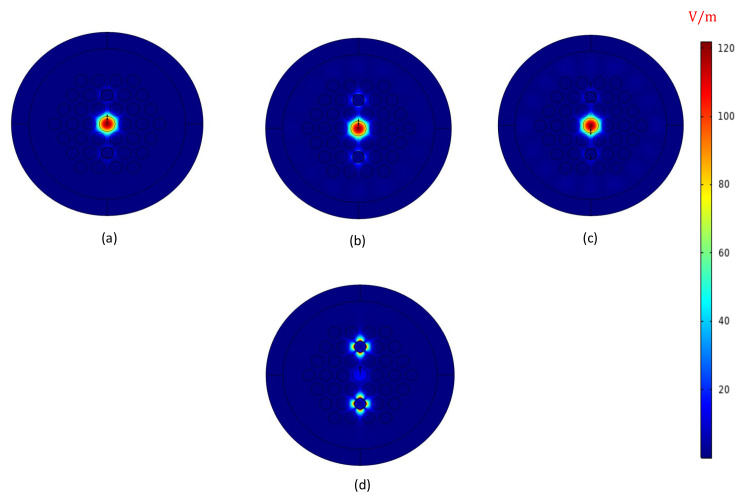
Electric field distributions of the HC-PCF-based SPR sensor for fundamental core mode at (**a**) 1200 nm, (**b**) 1244 nm (RPW), (**c**) 1300 nm and plasmonic mode at (**d**) 1244 nm of $n_{a}$ = 1.45.

**Figure 4:**
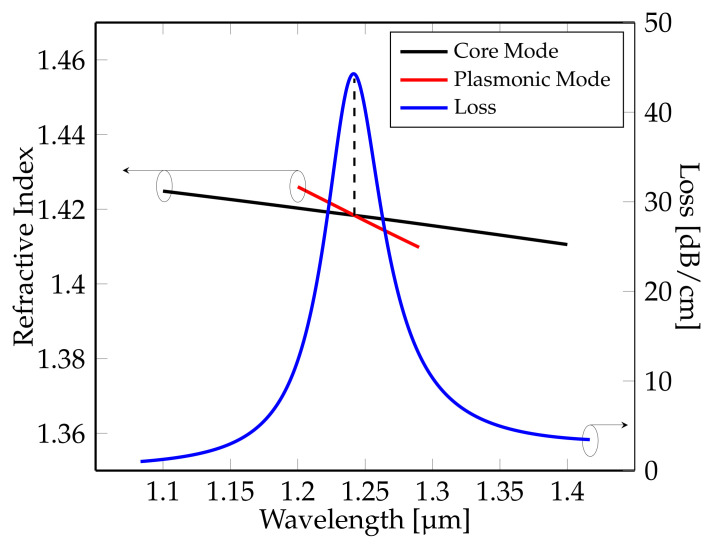
Dispersion relation of the fundamental core mode, plasmonic mode and confinement loss for analyte with RI of $n_{a}$ = 1.45.

Figure 3 shows the electric field distributions of the fundamental core mode for three wavelengths; 1200 nm, 1244 nm (RPW), 1300 nm and plasmonic mode (or surface plasmon mode) for the phase-matching point or RPW ( 1244 nm). The characteristics of fundamental core mode and plasmonic mode and confinement loss at phase-matching point or RPW, are illustrated in Figure 4. The dotted line in Figure 4 indicates the point where the fundamental core mode strongly couples with the plasmonic mode. This point as shown in the diagram corresponds to the peak of the confinement loss.

## 4. Modelling of RPW Equation(s) Using DoE for HC-PCF Sensor Tolerance Studies

Factorial design and Response Surface Methodology are the two types of DoE methods used in this research work for the statistical model derivation of RPW equation(s) with respect to the structural parameters of the HC-PCF-based SPR sensor across different tolerance levels.

### 4.1. DoE Factorial Design

Factorial design is expressed in the form of a first order polynomial (linear) equation [33]:

(5) $$Y=\beta_{0}+\sum_{i=1}^{k}\beta_{i}x_{i}+\epsilon$$

where Y is the response (RPW), *k* is the number of parameters (k=4 PCF structural parameters that correspond to air-hole diameter ($x_{1}$), pitch ($x_{2}$), gold layer thickness ($x_{3}$) and core diameter ($x_{4}$)), ϵ is the residual error. $\beta_{0}$, $\beta_{i}$ are offset constant and linear term coefficient, respectively. The offset constant which is the initial constant of the linear model Equation (5) is the average value of the response (RPW). The method of least squares is used to estimate the β coefficients [33]. Defining Y as a vector of length $n=2^{k}$ made of the RPW values calculated from n runs of FEM simulations as response, Equation (5) in matrix form is written as:

(6) Y=Dβ

where D is the matrix of size n×(k+1) constructed with the offset constant coefficient and different levels of parameters. β is a vector of length k+1 that consists of the offset constant $\beta_{0}$ and linear term coefficients $\beta_{i}$ to be calculated. To solve for β, Equation (6) is either expressed as $\beta =D^{-}Y\;\mathrm{or}\;\beta =D^{+}Y$, where $D^{-}$ is the inverse of D if D is a square matrix, otherwise the pseudo-inverse $D^{+}$ will be computed. Pseudo-inverse is calculated as: $D^{+}={\left(D^{T}D\right)}^{-1}D^{T}$, where $D^{T}$ is the transpose of D and ${\left(D^{T}D\right)}^{-1}$ is the inverse of $D^{T}D$. Finally, the calculated values for β are to be substituted in Equation (5) to develop the linear factorial design model for the RPW of the PCF-SPR sensor.

For the first tolerance level of ±2% variations in the HC-PCF sensor structural parameters variations considered, a 2-level factorial design for the four structural parameters of the sensor (Figure 1) for sensing analyte material with RI = 1.45 is shown in Table 2. The Minitab statistical software is used to perform the statistical and numerical analysis for k=4 parameters corresponding to $n=2^{k}=2^{4}=16$ simulations (see Table 3).

The calculated RPW from 16 different FEM simulations runs with the ±2% variations in the designed values of the structural parameters for an analyte RI of 1.45 are shown in the sixth column of Table 3. The high (+2%) and low (−2%) levels for each structural parameter (Table 2) are used for the FEM simulations run (first column in Table 3) with air-hole diameter varied for every one ($2^{0}=1$) of them (second column in Table 3), pitch varied for every two ($2^{1}=2$) of them (third column in Table 3), gold layer thickness varied for every four ($2^{2}=4$) of them (fourth column in Table 3) and core diameter varied for every eight ($2^{3}=8$) of them (fifth column in Table 3).

Factorial design is used for the preparation of the matrix D in Equation (6). Using the structure parameters for the D matrix and the values for RPW (response Y vector from last column) in Table 3, Equation (6) can be written as:

(7) $$Y=\left[\begin{array}{c} 1236\\ 1236\\ 1250\\ 1258\\ 1230\\ 1231\\ 1245\\ 1252\\ 1235\\ 1236\\ 1250\\ 1257\\ 1230\\ 1231\\ 1244\\ 1252 \end{array}\right];\;D=\left[\begin{array}{ccccc} 1 & 1.372 & 1.96 & 49 & 1.764\\ 1 & 1.428 & 1.96 & 49 & 1.764\\ 1 & 1.372 & 2.04 & 49 & 1.764\\ 1 & 1.428 & 2.04 & 49 & 1.764\\ 1 & 1.372 & 1.96 & 51 & 1.764\\ 1 & 1.428 & 1.96 & 51 & 1.764\\ 1 & 1.372 & 2.04 & 51 & 1.764\\ 1 & 1.428 & 2.04 & 51 & 1.764\\ 1 & 1.372 & 1.96 & 49 & 1.836\\ 1 & 1.428 & 1.96 & 49 & 1.836\\ 1 & 1.372 & 2.04 & 49 & 1.836\\ 1 & 1.428 & 2.04 & 49 & 1.836\\ 1 & 1.372 & 1.96 & 51 & 1.836\\ 1 & 1.428 & 1.96 & 51 & 1.836\\ 1 & 1.372 & 2.04 & 51 & 1.836\\ 1 & 1.428 & 2.04 & 51 & 1.836 \end{array}\right];\;\beta =\left[\begin{array}{c} \beta_{0}\\ \beta_{1}\\ \beta_{2}\\ \beta_{3}\\ \beta_{4} \end{array}\right]$$

Using the calculated value of β for the 2-level linear model (5), we derive:

(8) $$\mathrm{RPW}=835.8+73.7x_{1}+223.4x_{2}-2.688x_{3}-5.2x_{4}$$

where $x_{1}$, $x_{2}$, $x_{3}$ and $x_{4}$ represent the 4 HC-PCF-based SPR sensor parameters under consideration: air-hole diameter, pitch, gold layer thickness and core diameter, respectively.

**Figure 5:**
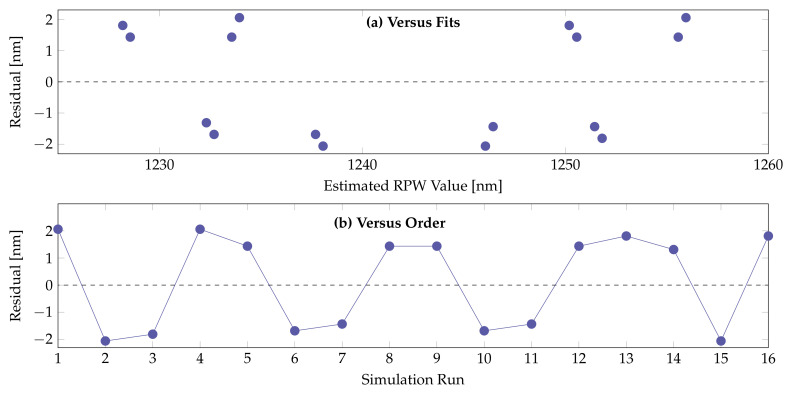
Residual analysis plots of the factorial design model for RPW: (**a**) Residual versus estimated RPW value scatter diagram and (**b**) Residual versus Simulation run scatter diagram.

Residual analysis was carried out to confirm the effectiveness of the derived linear model. In Figure 5a,b, the residual errors (blue coloured circled marks) are plotted against the estimated RPW values and number of FEM simulation runs. The statistical model equation (Equation (8)) derived for the RPW using DoE factorial method provides only the direct linear relationship of the 4 structural parameters influencing the sensing performance. For the derivation of the statistical model for that RPW that includes the nonlinear terms and inter-connected structural parameters influencing terms, we have to utilise the Response Surface Methodology available in DoE.

### 4.2. DoE Response Surface Methodology

The Response Surface Methodology (RSM) was introduced by Box and Wilson [26] to derive the nonlinear statistical model equations to estimate the response of interest that will include the higher-order and interplay effects of the parameters. The RSM statistical method used in our work to perform this DoE procedure is known as central composite design (CCD) [34,35]. This statistical procedure utilises a centre point to design a quadratic model for the RPW response of the HC-PCF-based SPR sensor. The model is expressed in **the form of a second-order quadratic equation** [34]:

(9) $$Y=\beta_{0}+\sum_{i=1}^{k}\beta_{i}x_{i}+\sum_{i=1}^{k}\beta_{\mathrm{ii}}x_{i}^{2}+\;\sum_{\begin{array}{c} \;\\ i,j=1\\ \;\\ i\neq j \end{array}}^{k}\;\beta_{\mathrm{ij}}x_{i}x_{j}+\epsilon$$

where $\beta_{0}$, $\beta_{i}$, $\beta_{\mathrm{ii}}$ and $\beta_{\mathrm{ij}}$ represent constant, linear, intra-quadratic and inter-quadratic terms coefficients, respectively. Thus, for the CCD procedure, the vector β length becomes ${}^{k+2}\;\;C_{2}$. In addition, Y becomes a vector of length ${}^{k+2}\;\;C_{2}$ to be constructed with the FEM simulations RPW (response) values. Consequently, D becomes a matrix of size $n\times {}^{k+2}\;\;C_{2}$ constructed with the offset constant coefficient and different levels of parameters in sequence of linear, intra-quadratic and inter-quadratic parameters values. Also for CCD, $n=2^{k}+2k+1$.

In the CCD procedure, the 2 levels (maximum and minimum) used for factorial analysis are increased to 5 levels. The centre point is introduced as well as the embedded fractional factorial levels.

Using the data in Table 4, statistical analysis and numerical analysis for k=4 parameters corresponding to $n=2^{4}+2\times 4+1=25$ scenarios are tested using the Minitab statistical software. The variations of +1% and −1% levels for the structural parameters are same as described in Table 3 for factorial design. As required, more FEM simulations runs (rows 17–25) are carried out using the variations of +2%, −2% and 0% levels (Table 4) for the structural parameters as depicted in Table 5.

Using the structure parameters for the D matrix and the values for RPW (response Y vector from last column) in Table 5, Equation (6) can be written as:

(10) $$Y=\left[\begin{array}{c} 1240\\ 1241\\ 1248\\ 1250\\ 1237\\ 1238\\ 1245\\ 1248\\ 1240\\ 1240\\ 1248\\ 1250\\ 1237\\ 1238\\ 1245\\ 1248\\ 1241\\ 1244\\ 1234\\ 1252\\ 1246\\ 1241\\ 1244\\ 1243\\ 1244 \end{array}\right];\;D=\left[\begin{array}{cccccccc} 1 & 1.386 & 1.98 & 49.5 & 1.782 & 1.921 & ... & 88.209\\ 1 & 1.414 & 1.98 & 49.5 & 1.782 & 1.9994 & ... & 88.209\\ 1 & 1.386 & 2.02 & 49.5 & 1.782 & 1.921 & ... & 88.209\\ 1 & 1.414 & 2.02 & 49.5 & 1.782 & 1.9994 & ... & 88.209\\ 1 & 1.386 & 1.98 & 50.5 & 1.782 & 1.921 & ... & 89.991\\ 1 & 1.414 & 1.98 & 50.5 & 1.782 & 1.9994 & ... & 89.991\\ 1 & 1.386 & 2.02 & 50.5 & 1.782 & 1.921 & ... & 89.991\\ 1 & 1.414 & 2.02 & 50.5 & 1.782 & 1.9994 & ... & 89.991\\ 1 & 1.386 & 1.98 & 49.5 & 1.818 & 1.921 & ... & 89.991\\ 1 & 1.414 & 1.98 & 49.5 & 1.818 & 1.9994 & ... & 89.991\\ 1 & 1.386 & 2.02 & 49.5 & 1.818 & 1.921 & ... & 89.991\\ 1 & 1.414 & 2.02 & 49.5 & 1.818 & 1.9994 & ... & 89.991\\ 1 & 1.386 & 1.98 & 50.5 & 1.818 & 1.921 & ... & 91.809\\ 1 & 1.414 & 1.98 & 50.5 & 1.818 & 1.9994 & ... & 91.809\\ 1 & 1.386 & 2.02 & 50.5 & 1.818 & 1.921 & ... & 91.809\\ 1 & 1.414 & 2.02 & 50.5 & 1.818 & 1.9994 & ... & 91.809\\ 1 & 1.372 & 2 & 50 & 1.8 & 1.8824 & ... & 90\\ 1 & 1.428 & 2 & 50 & 1.8 & 2.0392 & ... & 90\\ 1 & 1.4 & 1.96 & 50 & 1.8 & 1.96 & ... & 90\\ 1 & 1.4 & 2.04 & 50 & 1.8 & 1.96 & ... & 90\\ 1 & 1.4 & 2 & 49 & 1.8 & 1.96 & ... & 88.2\\ 1 & 1.4 & 2 & 51 & 1.8 & 1.96 & ... & 91.8\\ 1 & 1.4 & 2 & 50 & 1.764 & 1.96 & ... & 88.2\\ 1 & 1.4 & 2 & 50 & 1.836 & 1.96 & ... & 91.8\\ 1 & 1.4 & 2 & 50 & 1.8 & 1.96 & ... & 90 \end{array}\right];\;\beta =\left[\begin{array}{c} \beta_{0}\\ \beta_{1}\\ \beta_{2}\\ \beta_{3}\\ \beta_{4}\\ \beta_{11}\\ \beta_{22}\\ \beta_{33}\\ \beta_{44}\\ \beta_{12}\\ \beta_{13}\\ \beta_{14}\\ \beta_{23}\\ \beta_{24}\\ \beta_{34} \end{array}\right]$$

where the D matrix values are calculated as 1(offset), *d*, Λ, $t_{g}$, $d_{c}$, $d^{2}$, $\Lambda^{2}$, $t_{g}^{2}$, $d_{c}^{2}$, dΛ, $dt_{g}$, $dd_{c}$, $\Lambda t_{g}$, $\Lambda d_{c}$ and $t_{g}d_{c}$. Table 5 shows the RPWs from 25 different FEM simulation runs with the designed structural parameters for an analyte RI of $n_{a}$ = 1.45. Using the calculated value of β for the 5-level quadratic model Equation (9), we derive:

(11) $$\begin{array}{cc} \mathrm{RPW} & =861+949x_{1}-402x_{2}-27.6x_{3}+688x_{4}-1754x_{1}^{2}-547x_{2}^{2}-0.375x_{3}^{2}-289x_{4}^{2}\\ \; & \;+1562x_{1}x_{2}+26.79x_{1}x_{3}-248x_{1}x_{4}+6.25x_{2}x_{3}+174x_{2}x_{4}+6.94x_{3}x_{4} \end{array}$$

**Figure 6:**
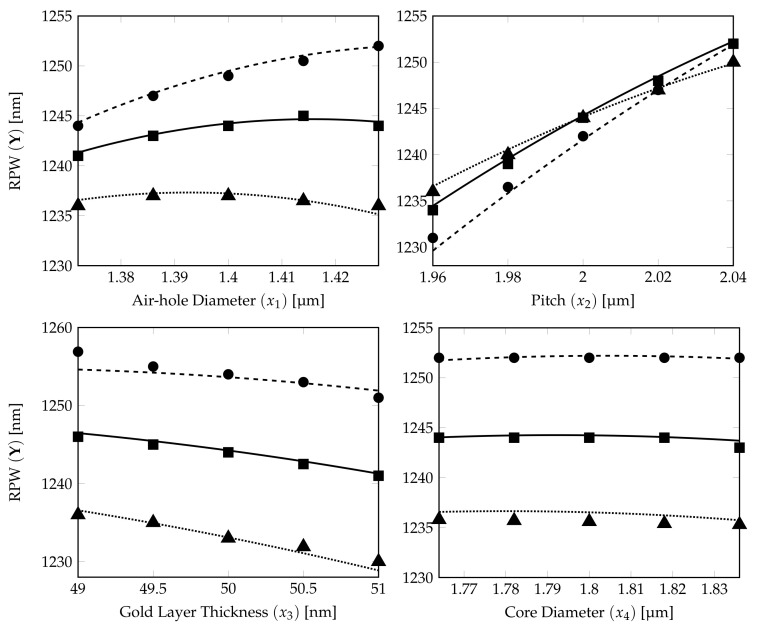
Effects of four factors (air-hole diameter, pitch, gold layer thickness and core diameter of analyte) on RPW curves generated from quadratic CCD ±2% model. The dotted, solid and dashed curves show the RPW calculated from the ±2% DoE model (11) with three parameters (expected target parameter on *x*-axis) fixed at their own tolerance levels, −2%, 0% and +2%, respectively. The RPW marks (▴⟶−2%, ▪⟶ 0% and •⟶+2%) are calculated from corresponding FEM simulations.

The relationship between the DoE RPW model obtained in Equation (11) and the RPW-FEM simulations for the different structural parameters are shown in Figure 6.

The 15 RPW values calculated from FEM simulations are plotted on the corresponding RPW model (11) curves (5 marks for each characteristic curve) in Figure 6. It shows that the prediction from the derived DoE Equation (11) is a very good estimate. The dotted, solid and dashed curves show the RPWs calculated from DoE model (11) with ±2% variations for the PCF parameter represented by the *x*-axis label and with three parameters fixed at their own tolerance levels, −2%, 0% and +2%, respectively (see Table 4). The ▪, • and ▴ marks show the corresponding RPW values from the FEM simulation results for DoE model accuracy verification of those dotted, solid and dashed lines. 

From the subplots, except the one for the pitch, the curves for the RPW in the air-hole diameter, gold layer thickness and core diameter plots are separated from each other without any crossing. The RPW lines in the air-hole diameter subplot are having more curvature, which shows that air-hole diameter has a slight quadratic effect influence for the RPW estimation for the ±2% DoE model. For the gold layer thickness and core diameter subplots, the equally spaced straight lines show that these two parameters are dominated only by the linear effects on the derived RPW for the ±2% DoE model.

We can conclude that pitch plays the most important role (nonlinear influence) in determining the RPW for the ±2% DoE model. However, there is possibility for the characteristics from the air-hole diameter plot to have crosspoint(s) at higher tolerance levels as weak quadratic nonlinear effect is depicted at ±2% variations in the values for the structural parameters. To investigate further on the tolerance studies, we have performed the DoE statistical modelling for two higher level variations, respectively, for ±5% and ±10% ranges.

- **DoE Model with ±5%**

The DoE quadratic model of a higher tolerance (±5%) is investigated to further find the possible best fit model for fabrication purposes. The full details for the FEM simulation of the HC-PCF-SPR sensor with (±5%) fabrication tolerance are provided in the following and the DoE equation derived is of same functional form like the ±2% variations, but with a different set of the constant and coefficients.

The DoE modelling calculation of the HC-PCF-SPR sensor with ±5% tolerance is presented in detail. Table 6 shows the 5 levels design of four parameters with ±5% fabrication tolerance.

Using the data in Table 6, statistical analysis and numerical analysis for 25 scenarios are tested using the Minitab statistical software.

Using the structure parameters for the D matrix and for RPW (response Y vector from last column) in Table 7, Equation (6) can be written similar to (10). Table 7 shows the RPWs from 25 different FEM simulation runs with the designed structural parameters for an analyte RI of $n_{a}=1.45$. For the calculated value of β for the 5-level quadratic model (9), we derive:

(12) $$\begin{array}{cc} \mathrm{RPW} & =711+401x_{1}+258x_{2}-17.23x_{3}+251x_{4}-1113.9x_{1}^{2}-545.8x_{2}^{2}+0.087x_{3}^{2}-56.6x_{4}^{2}\\ \; & \;+1464.3x_{1}x_{2}-1.43x_{1}x_{3}-39.7x_{1}x_{4}+3x_{2}x_{3}-27.8x_{2}x_{4}+1.11x_{3}x_{4} \end{array}$$

**Figure 7:**
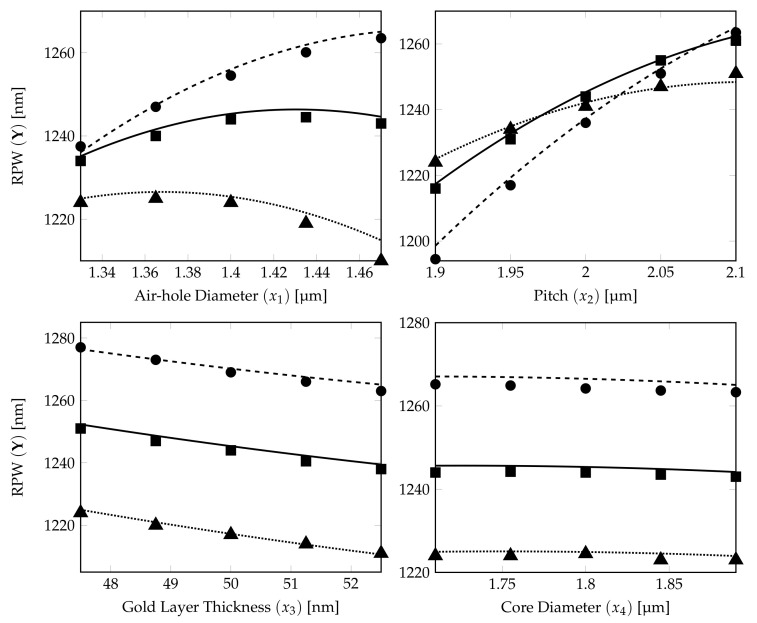
Effects of four factors (air-hole diameter, pitch, gold layer thickness and core diameter of analyte) on RPW curves generated from quadratic CCD ±5% model. The dotted, solid and dashed curves show the RPW calculated from the ±5% DoE model (12) with three parameters (expected target parameter on *x*-axis) fixed at their own tolerance levels, −5%, 0% and +5%, respectively. The RPW marks (▴⟶−5%, *▪*⟶ 0% and ⟶+5%) are calculated from corresponding FEM simulations.

The relationship between the DoE RPW model obtained in Equation (12) and the RPW-FEM simulations for the different structural parameters are shown in Figure 7.

The 15 RPW values calculated from FEM simulations are plotted on the corresponding RPW model (12) curves (5 marks for each characteristics curve) in Figure 7. It shows that the prediction from the derived DoE Equation (12) is a very good estimate. The dotted, solid and dashed curves show the RPWs calculated from DoE model (12) with ±5% variations for the PCF parameter represented by the *x*-axis label and with three parameters fixed at their own tolerance levels, −5%, 0% and +5%, respectively (see Table 6). The ▪, • and ▴ marks show the corresponding RPW values from the FEM simulation results for DoE model accuracy verification of those dotted, solid and dashed lines.

From the subplots, except the air-hole diameter and pitch in this case, the curves for the RPW in the gold layer thickness and core diameter plots are still separated from each other without any crossing. The RPW lines in the air-hole diameter subplot are having more curvature and dashed line (+5%) and solid line (0%) will have a crossing for lower air-hole diameter which shows that air-hole diameter has stronger quadratic effect influence for the RPW estimation in the ±5% DoE model compared with the ±2% DoE model.

We can summarise that besides pitch, air-hole diameter plays also one of the important roles (nonlinear influence) in determining the RPW for the ±5% DoE model. The possibility for the characteristics from the air-hole diameter plot to have crosspoint(s) at higher tolerance levels as weak quadratic nonlinear effect can be expected at ±5% variations in the values for the structural parameters. However, this needs further confirmation. To investigate further on the tolerance studies, we have performed the DoE statistical modelling for another higher level of variation (±10% range).

- **DoE Model with ±10%**

The DoE quadratic model of a higher tolerance (±10%) is investigated to further find the possible best fit model for fabrication purposes. Similar to the previous case for the ±5% tolerance study, the full details for the FEM simulation of the HC-PCF-SPR sensor with (±10%) fabrication tolerance are provided in the following and the DoE equation derived is of same functional form like the previous ±2% and ±5% fabrication tolerances, but with a different set of the constant and coefficients.

The DoE modelling calculation of the HC-PCF-SPR sensor with ±10% tolerance is presented in detail. Table 8 shows the 5-level design of four parameters with ±10% fabrication tolerance.

Using the data in Table 8, statistical analysis and numerical analysis for 25 scenarios are tested using the Minitab statistical software.

Using the structure parameters for the D matrix and the values for RPW (response Y vector from last column) in Table 9, Equation (6) can be written similar to (10). Table 9 shows the RPWs from 25 different FEM simulation runs with the designed structural parameters for an analyte RI of $n_{a}=1.45$. For the calculated value of β for the 5-level quadratic model (9), we derive:

(13) $$\begin{array}{cc} \mathrm{RPW} & =641+295x_{1}+370x_{2}-9.68x_{3}+75x_{4}-1090.6x_{1}^{2}-546.9x_{2}^{2}+0.085x_{3}^{2}-11.6x_{4}^{2}\\ \; & \;+1473.2x_{1}x_{2}-2.5x_{1}x_{3}-9.9x_{1}x_{4}+0.75x_{2}x_{3}-20.8x_{2}x_{4}+0.28x_{3}x_{4} \end{array}$$

**Figure 8:**
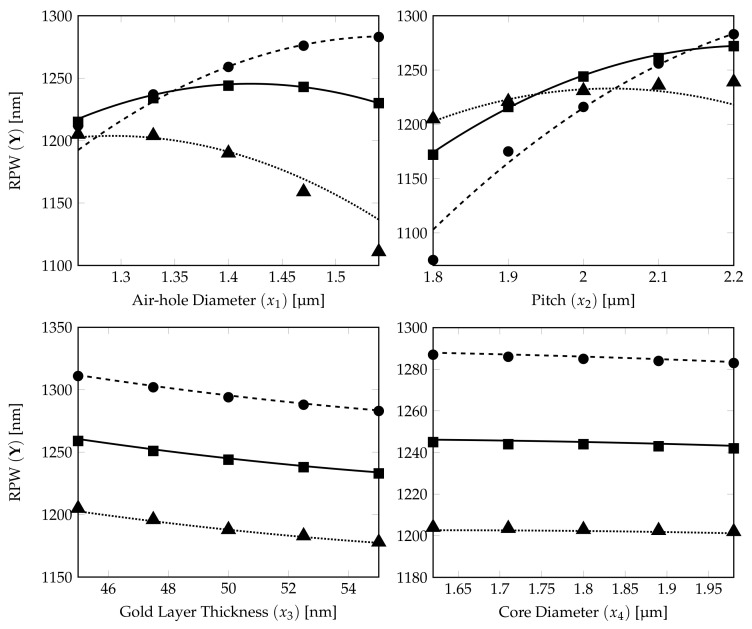
Effects of four factors (air-hole diameter, pitch, gold layer thickness and core diameter of analyte) on RPW curves generated from quadratic CCD ±10% model. The dotted, solid and dashed curves show the RPW calculated from the ±2% DoE model (13) with three parameters (expected target parameter on *x*-axis) fixed at their own tolerance levels, −10%, 0% and +10%, respectively. The RPW marks (▴⟶−10%, ▪⟶ 0% and •⟶+10%) are calculated from corresponding FEM simulations.

The relationship between the DoE RPW models obtained in Equation (13) and the RPW-FEM simulations for the different structural parameters are shown in Figure 8.

The 15 RPW values calculated from FEM simulations are plotted on the corresponding RPW model (13) curves (5 marks for each characteristics curve) in Figure 8. It shows that the prediction from the derived DoE Equation (13) is a very good estimate. The dotted, solid and dashed curves show the RPWs calculated from DoE model (13) with ±10% variations for the PCF parameter represented by the *x*-axis label and with three parameters fixed at their own tolerance levels, −10%, 0% and +10%, respectively (see Table 8). The ▪, • and ▴ marks show the corresponding RPW values from the FEM simulation results for DoE model accuracy verification of those dotted, solid and dashed lines.

From the subplots, except the air-hole diameter and pitch in this case, the curves for the RPW in the gold layer thickness and core diameter plots are separated from each other without any crossing as in the previous case. The RPW lines in the air-hole diameter subplot are having more curvature and dashed line (+5%) and solid line (0%) now have two crossings in lower air-hole diameter region which confirms that the air-hole diameter has strong quadratic effect influence for the RPW estimation in the ±10% DoE model compared with the ±2% and ±5% DoE model.

We can conclude that pitch and air-hole diameter not only play important roles (nonlinear influence) in determining the RPW for the ±5% DoE model, but also they both exhibit the characteristics in the subplots to have crosspoints at higher tolerance levels as quadratic nonlinear effect can be expected at ±10% variations in the values for the structural parameters. The level of quadratic nonlinear effect caused by air-hole diameter increases as the tolerance level increases. Moreover, the crosspoints and the strong quadratic nonlinear effect from air-hole diameter on the RPW cannot be easily seen/inferred for a DoE model with low tolerance.

Comparison between the coefficients of the three DoE models with different fabrication tolerances are listed in Table 10. High absolute values for $\beta_{1}$, $\beta_{11}$, $\beta_{2}$ and $\beta_{22}$ (first- and second-order coefficients for air-hole diameter and pitch, respectively) can be found for all three models. This matches with the conclusion that those two structure parameters play the dominating roles for the determination of RPW. It should be noted that for quadratic coefficient of pitch ($\beta_{22}$), it kept almost unchanged for all three tolerances with high absolute values, whereas the second-order coefficient of air-hole diameter ($\beta_{11}$) increases drastically in the negative direction. These characteristics of the coefficients confirm the conclusions arrived from the behaviour noticed from the graphs for the quadratic effect behaviour of pitch and air-hole diameter on the RPW. With respect to the coefficients $\beta_{4}$ and $\beta_{44}$ corresponding to the first-order and second-order terms for the gold layer thickness, even though, both the absolute values and changes are significant, the role played by this parameter has barely any quadratic effect on the determination of the RPW as the unit for the metal coating (nm) is three orders lower than the other three parameters (μm). Therefore, the quadratic effects for parameters of gold layer thickness and core diameter with small coefficients are not crucial for the determination of the RPW, which again confirms the findings from the graphs.

**Figure 9:**
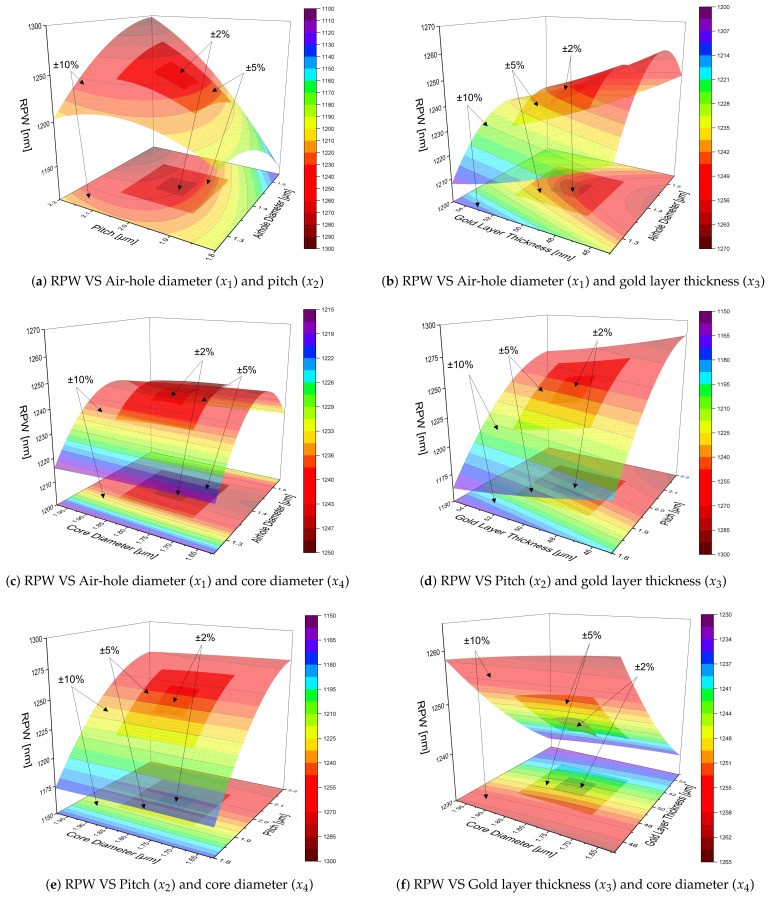
Response surface (3D) and contour (2D) plots of RPW with respect to the interaction of any two among the four structural parameters (air-hole diameter, pitch, gold layer thickness and core diameter) for ±2%, ±5% and ±10% tolerance levels. Different levels of transparency for the surface and contour plots are set for identification as 0% (for ±2%), 25% (for ±5%), 50% (for ±10%).

Figure 9 illustrates the RPW response surface (3D) and contour (2D) plots for the interaction of any two among the four structural parameters of the proposed HC-PCF SPR sensor, corresponding to air-hole diameter, pitch, gold layer thickness and core diameter tolerance levels of ±2%, ±5% and ±10%. From these plots, it is noticeable that the ±10% DoE model can provide overall characteristics of the RPW variations with any two structural parameters and it can represent most of the parametric ranges that are provided by the ±2% and ±5% DoE models. Thus, Equation (13) corresponding to ±10% DoE model is proved to be the general RPW equation that also includes ±2% and ±5% tolerance level cases. However, it should be noted that finer detail differences can still be found between the surfaces for different tolerance levels due to the model residual error ϵ defined in Equation (9). Particularly, this is observed in ±5% and ±10% surfaces in Figure 9c and ±2% and ±5% surfaces in Figure 9f. Hence, the proposed statistical modelling of the PCF-SPR sensor response (RPW in this work) for the structural parametric range to be developed is based on requirements such as the overall general characteristics for larger range variations or finer details for particular small range variations.

Results from the DoE models for three tolerance levels, ±2% (Equation (11)), ±5% (Equation (12)) and ±10% (Equation (13)) as illustrated in Figure 6, Figure 7 and Figure 8, show that DoE model can present good predictions and exhibit linear or quadratic influences on the determination of the RPW of HC-PCF-based SPR sensors. For the proposed HC-PCF sensor, pitch has been discovered to have the most quadratic effect in determining the RPW of this sensor among all three tolerance levels. Gold layer thickness and core diameter were discovered to have very steady linear effects as tolerance increases. This means that during the fabrication of HC-PCF-based SPR sensors, structural variations of the gold layer thickness and core diameter from tolerances ±2%, ±5% to ±10% will not affect the stability of RPWs and, hence, the sensing performances of the sensor. On the other hand, the air-hole diameter will cause comparatively significant quadratic effect on the RPW of HC-PCF-based SPR sensors for high fabrication tolerance level (±10%), moderate quadratic effect with typical fabrication tolerance level (±5%) and the quadratic effect can hardly be found for low fabrication tolerance level (±2%).

## 5. Conclusions

In this paper, we presented a statistical approach of determining the influence of the optical fibre structural design parameters on the sensing performance of HC-PCF-based SPR sensors at three tolerance levels (±2%, ±5% and ±10%). Two statistical DoE methods, factorial design and RSM design, were presented in detail to derive the model equation(s) for the RPW in terms of the structural parameters of the HC-PCF-based SPR sensor for the tolerance studies. The whole work begun with FEM simulation for the sensor design which could achieve a maximum sensitivity of 5600 nm/RIU for analytes detection with RI range of 1.43 to 1.49. Then, factorial design and CCD were demonstrated for the modelling and calculations procedures. The DoE models based on FEM simulation results for RPW estimation with three tolerance levels were analysed and compared in detail.

Results from the investigation on three models show that structural parameters (air-hole diameter, pitch, gold layer thickness and core diameter) have linear or quadratic effects on the RPW determination for the sensing process. A clear understanding of the individual parameters influence in determining the RPW changes was presented with the DoE methodology combined with a few FEM simulation results which would not be feasible with numerical computations only. For the proposed HC-PCF sensor, pitch was the most significant parameter for the quadratic effect influence on RPW and gold layer thickness and core diameter are the least contributing parameters. The significance of quadratic effect influence for air-hole diameter was found to be proportional to the increase of fabrication tolerance level. Due to the amount of time and computational resource saved by using the proposed research methodology on tolerance study, we envisage that this novel statistical model derivation of sensing performance parameters can be applied in fibre sensor fabrication industry to improve efficiency and lower cost. 

## Figures and Tables

**Table 1:** Sensing performance of the HC-PCF-based SPR sensor in the RI range from 1.43 to 1.49.

S/N	Analyte RI	$n_{\mathrm{eff}}$	Peak Loss [dB/cm]	RPW [nm]	Sensitivity [nm/RIU]
1	1.43	1.3986	166.039	1355	N/A
2	1.44	1.4083	86.192	1299	5600
3	1.45	1.4183	43.663	1244	5500
4	1.46	1.4284	20.857	1189	5500
5	1.47	1.4387	9.262	1137	5200
6	1.48	1.4491	3.800	1088	4900
7	1.49	1.4596	1.454	1044	4400

**Table 2:** Two-level Four Parameters (±2% variations) and their values for DoE Factorial Analysis.

PCF Parameters	−2%	+2%
Air-hole diameter, *d* [μm]	1.372	1.428
Pitch, Λ [μm]	1.96	2.04
Gold layer thickness, $t_{g}$ [nm]	49	51
Core diameter, $d_{c}$ [μm]	1.764	1.836

**Table 3:** FEM Simulations for Two-Level 4-Parameter (±2% variations) Factorial Analysis.

Simulation Run	Air-Hole Diameter *d* [μm]	Pitch *Λ* [μm]	Gold Layer Thickness *t_g_* [nm]	Core Diameter *d_c_* [μm]	RPW [nm]
1	1.372 (−2%)	1.96 (−2%)	49 (−2%)	1.764 (−2%)	1236
2	1.428 (+2%)	1.96 (−2%)	49 (−2%)	1.764 (−2%)	1236
3	1.372 (−2%)	2.04 (+2%)	49 (−2%)	1.764 (−2%)	1250
4	1.428 (+2%)	2.04 (+2%)	49 (−2%)	1.764 (−2%)	1258
5	1.372 (−2%)	1.96 (−2%)	51 (+2%)	1.764 (−2%)	1230
6	1.428 (+2%)	1.96 (−2%)	51 (+2%)	1.764 (−2%)	1231
7	1.372 (−2%)	2.04 (+2%)	51 (+2%)	1.764 (−2%)	1245
8	1.428 (+2%)	2.04 (+2%)	51 (+2%)	1.764 (−2%)	1252
9	1.372 (−2%)	1.96 (−2%)	49 (−2%)	1.836 (+2%)	1235
10	1.428 (+2%)	1.96 (−2%)	49 (−2%)	1.836 (+2%)	1236
11	1.372 (−2%)	2.04 (+2%)	49 (−2%)	1.836 (+2%)	1250
12	1.428 (+2%)	2.04 (+2%)	49 (−2%)	1.836 (+2%)	1257
13	1.372 (−2%)	1.96 (−2%)	51 (+2%)	1.836 (+2%)	1230
14	1.428 (+2%)	1.96 (−2%)	51 (+2%)	1.836 (+2%)	1231
15	1.372 (−2%)	2.04 (+2%)	51 (+2%)	1.836 (+2%)	1244
16	1.428 (+2%)	2.04 (+2%)	51 (+2%)	1.836 (+2%)	1252

**Table 4:** Five-level Four Parameters (±2% variations) and Their Values for DoE CCD Analysis.

PCF Parameters	−2%	−1%	0%	+1%	+2%
Air-hole diameter, *d* [μm]	1.372	1.386	1.4	1.414	1.428
Pitch, Λ [μm]	1.96	1.98	2	2.02	2.04
Gold layer thickness, $t_{g}$ [nm]	49	49.5	50	50.5	51
Core diameter, $d_{c}$ [μm]	1.764	1.782	1.8	1.818	1.836

**Table 5:** FEM Simulations for 5-Level 4-Parameter (±2% variations) DoE CCD Analysis.

Simulation Run	Air-Hole Diameter *d* [μm]	Pitch *Λ* [μm]	Gold Layer Thickness *t_g_* [nm]	Core Diameter $d_{c}$ [	RPW [nm]
1	1.386 (−1%)	1.98 (−1%)	49.5 (−1%)	1.782 (−1%)	1240
2	1.414 (+1%)	1.98 (−1%)	49.5 (−1%)	1.782 (−1%)	1241
3	1.386 (−1%)	2.02 (+1%)	49.5 (−1%)	1.782 (−1%)	1248
4	1.414 (+1%)	2.02 (+1%)	49.5 (−1%)	1.782 (−1%)	1250
5	1.386 (−1%)	1.98 (−1%)	50.5 (+1%)	1.782 (−1%)	1237
6	1.414 (+1%)	1.98 (−1%)	50.5 (+1%)	1.782 (−1%)	1238
7	1.386 (−1%)	2.02 (+1%)	50.5 (+1%)	1.782 (−1%)	1245
8	1.414 (+1%)	2.02 (+1%)	50.5 (+1%)	1.782 (−1%)	1248
9	1.386 (−1%)	1.98 (−1%)	49.5 (−1%)	1.818 (+1%)	1240
10	1.414 (+1%)	1.98 (−1%)	49.5 (−1%)	1.818 (+1%)	1240
11	1.386 (−1%)	2.02 (+1%)	49.5 (−1%)	1.818 (+1%)	1248
12	1.414 (+1%)	2.02 (+1%)	49.5 (−1%)	1.818 (+1%)	1250
13	1.386 (−1%)	1.98 (−1%)	50.5 (+1%)	1.818 (+1%)	1237
14	1.414 (+1%)	1.98 (−1%)	50.5 (+1%)	1.818 (+1%)	1238
15	1.386 (−1%)	2.02 (+1%)	50.5 (+1%)	1.818 (+1%)	1245
16	1.414 (+1%)	2.02 (+1%)	50.5 (+1%)	1.818 (+1%)	1248
17	1.372 (−2%)	2 (0%)	50 (0%)	1.8 (0%)	1241
18	1.428 (+2%)	2 (0%)	50 (0%)	1.8 (0%)	1244
19	1.4 (0%)	1.96 (−2%)	50 (0%)	1.8 (0%)	1234
20	1.4 (0%)	2.04 (+2%)	50 (0%)	1.8 (0%)	1252
21	1.4 (0%)	2 (0%)	49 (−2%)	1.8 (0%)	1246
22	1.4 (0%)	2 (0%)	51 (+2%)	1.8 (0%)	1241
23	1.4 (0%)	2 (0%)	50 (0%)	1.764 (−2%)	1244
24	1.4 (0%)	2 (0%)	50 (0%)	1.836 (+2%)	1243
25	1.4 (0%)	2 (0%)	50 (0%)	1.8 (0%)	1244

**Table 6:** Five-level Four Parameters (±5% variations) and their values for DoE CCD Analysis.

PCF Parameters	−5%	−2.5%	0%	+2.5%	+5%
Air-hole diameter, *d* [μm]	1.33	1.365	1.4	1.435	1.47
Pitch, Λ [μm]	1.9	1.95	2	2.05	2.1
Gold layer thickness, $t_{g}$ [nm]	47.5	48.75	50	51.25	52.5
Core diameter, $d_{c}$ [μm]	1.71	1.755	1.8	1.845	1.89

**Table 7:** FEM Simulations for Five-Level 4-Parameter (±5% variations) CCD Analysis.

Simulation Run	Air-Hole Diameter *d* [μm]	Pitch *Λ* [μm]	Gold Layer Thickness *t_g_* [nm]	Core Diameter *d_c_* [μm]	RPW [nm]
1	1.365 (−2.5%)	1.95 (−2.5%)	48.75 (−2.5%)	1.755 (−2.5%)	1234
2	1.435 (+2.5%)	1.95 (−2.5%)	48.75 (−2.5%)	1.755 (−2.5%)	1234
3	1.365 (−2.5%)	2.05 (+2.5%)	48.75 (−2.5%)	1.755 (−2.5%)	1251
4	1.435 (+2.5%)	2.05 (+2.5%)	48.75 (−2.5%)	1.755 (−2.5%)	1261
5	1.365 (−2.5%)	1.95 (−2.5%)	51.25 (+2.5%)	1.755 (−2.5%)	1227
6	1.435 (+2.5%)	1.95 (−2.5%)	51.25 (+2.5%)	1.755 (−2.5%)	1227
7	1.365 (−2.5%)	2.05 (+2.5%)	51.25 (+2.5%)	1.755 (−2.5%)	1245
8	1.435 (+2.5%)	2.05 (+2.5%)	51.25 (+2.5%)	1.755 (−2.5%)	1255
9	1.365 (−2.5%)	1.95 (−2.5%)	48.75 (−2.5%)	1.845 (+2.5%)	1233
10	1.435 (+2.5%)	1.95 (−2.5%)	48.75 (−2.5%)	1.845 (+2.5%)	1233
11	1.365 (−2.5%)	2.05 (+2.5%)	48.75 (−2.5%)	1.845 (+2.5%)	1250
12	1.435 (+2.5%)	2.05 (+2.5%)	48.75 (−2.5%)	1.845 (+2.5%)	1260
13	1.365 (−2.5%)	1.95 (−2.5%)	51.25 (+2.5%)	1.845 (+2.5%)	1227
14	1.435 (+2.5%)	1.95 (−2.5%)	51.25 (+2.5%)	1.845 (+2.5%)	1226
15	1.365 (−2.5%)	2.05 (+2.5%)	51.25 (+2.5%)	1.845 (+2.5%)	1244
16	1.435 (+2.5%)	2.05 (+2.5%)	51.25 (+2.5%)	1.818 (+2.5%)	1254
17	1.330 (−5%)	2 (0%)	50 (0%)	1.800 (0%)	1234
18	1.470 (+5%)	2 (0%)	50 (0%)	1.800 (0%)	1243
19	1.4 (0%)	1.90 (−5%)	50 (0%)	1.800 (0%)	1216
20	1.4 (0%)	2.10 (+5%)	50 (0%)	1.800 (0%)	1261
21	1.4 (0%)	2 (0%)	47.50 (−5%)	1.800 (0%)	1251
22	1.4 (0%)	2 (0%)	52.50 (+5%)	1.800 (0%)	1238
23	1.4 (0%)	2 (0%)	50 (0%)	1.710 (−5%)	1244
24	1.4 (0%)	2 (0%)	50 (0%)	1.890 (+5%)	1243
25	1.4 (0%)	2 (0%)	50 (0%)	1.800 (0%)	1244

**Table 8:** Five-level Four Parameters (±10% variations) and their values for DoE CCD Analysis.

PCF Parameters	−10%	−5%	0%	+5%	+10%
Air-hole diameter, *d* [μm]	1.26	1.33	1.4	1.47	1.54
Pitch, Λ [μm]	1.8	1.9	2	2.1	2.2
Gold layer thickness, $t_{g}$ [nm]	45	47.5	50	52.5	55
Core diameter, $d_{c}$ [μm]	1.62	1.71	1.8	1.89	1.98

**Table 9:** FEM Simulations for Five-Level 4-Parameter (±10% variations) CCD Analysis.

Simulation Run	Air-Hole Diameter *d* [μm]	Pitch *Λ* [μm]	Gold Layer Thickness *t_g_* [nm]	Core Diameter *d_c_* [μm]	RPW [nm]
1	1.33 (−5%)	1.9 (−5%)	47.5 (−5%)	1.71 (−5%)	1224
2	1.47 (+5%)	1.9 (−5%)	47.5 (−5%)	1.71 (−5%)	1210
3	1.33 (−5%)	2.1 (+5%)	47.5 (−5%)	1.71 (−5%)	1252
4	1.47 (+5%)	2.1 (+5%)	47.5 (−5%)	1.71 (−5%)	1279
5	1.33 (−5%)	1.9 (−5%)	52.5 (+5%)	1.71 (−5%)	1211
6	1.47 (+5%)	1.9 (−5%)	52.5 (+5%)	1.71 (−5%)	1195
7	1.33 (−5%)	2.1 (+5%)	52.5 (+5%)	1.71 (−5%)	1239
8	1.47 (+5%)	2.1 (+5%)	52.5 (+5%)	1.71 (−5%)	1265
9	1.33 (−5%)	1.9 (−5%)	47.5 (−5%)	1.89 (+5%)	1223
10	1.47 (+5%)	1.9 (−5%)	47.5 (−5%)	1.89 (+5%)	1209
11	1.33 (−5%)	2.1 (+5%)	47.5 (−5%)	1.89 (+5%)	1250
12	1.47 (+5%)	2.1 (+5%)	47.5 (−5%)	1.89 (+5%)	1277
13	1.33 (−5%)	1.9 (−5%)	52.5 (+5%)	1.89 (+5%)	1210
14	1.47 (+5%)	1.9 (−5%)	52.5 (+5%)	1.89 (+5%)	1194
15	1.33 (−5%)	2.1 (+5%)	52.5 (+5%)	1.89 (+5%)	1238
16	1.47 (+5%)	2.1 (+5%)	52.5 (+5%)	1.89 (+5%)	1263
17	1.26 (−10%)	2 (0%)	50 (0%)	1.8 (0%)	1215
18	1.54 (+10%)	2 (0%)	50 (0%)	1.8 (0%)	1230
19	1.4 (0%)	1.8 (−10%)	50 (0%)	1.8 (0%)	1172
20	1.4 (0%)	2.2 (+10%)	50 (0%)	1.8 (0%)	1272
21	1.4 (0%)	2 (0%)	45 (−10%)	1.8 (0%)	1259
22	1.4 (0%)	2 (0%)	55 (+10%)	1.8 (0%)	1233
23	1.4 (0%)	2 (0%)	50 (0%)	1.62 (−10%)	1245
24	1.4 (0%)	2 (0%)	50 (0%)	1.98 (+10%)	1242
25	1.4 (0%)	2 (0%)	50 (0%)	1.8 (0%)	1244

**Table 10:** The coefficients of RPW model equations with tolerance levels corresponding to HC-PCF structural parameter value variations of ±2% (Equation (11)), ±5% (Equation (12)) and ±10% (Equation (13)).

	±2%	±5%	±10%	Characteristics Behaviour
$\beta_{0}$	861	711	641	decreasing with no sign change
$\beta_{1}$	949	401	295	decreasing with no sign change
$\beta_{2}$	−402	258	370	increasing with sign change
$\beta_{3}$	−27.6	−17.23	−9.68	increasing with no sign change
$\beta_{4}$	688	251	75	decreasing with no sign change
$\beta_{11}$	−1754	−1113.9	−1090.6	saturating with no sign change
$\beta_{22}$	−547	−545.8	−546.9	almost no change
$\beta_{33}$	−0.375	0.087	0.085	saturating with sign change
$\beta_{44}$	−289	−56.6	−11.6	increasing with no sign change
$\beta_{12}$	1562	1464.3	1473.2	saturating with no sign change
$\beta_{13}$	26.79	−1.43	−2.5	decreasing with no sign change
$\beta_{14}$	−248	−39.7	−9.9	increasing with no sign change
$\beta_{23}$	6.25	3	0.75	decreasing with no sign change
$\beta_{24}$	174	−27.8	−20.8	saturating with sign change
$\beta_{34}$	6.94	1.11	0.28	decreasing with no sign change
